# Genetic homogeneity of the invasive lionfish across the Northwestern Atlantic and the Gulf of Mexico based on Single Nucleotide Polymorphisms

**DOI:** 10.1038/s41598-018-23339-w

**Published:** 2018-03-22

**Authors:** R. Pérez-Portela, A. Bumford, B. Coffman, S. Wedelich, M. Davenport, A. Fogg, M. K. Swenarton, F. Coleman, M. A. Johnston, D. L. Crawford, M. F. Oleksiak

**Affiliations:** 1 Rosenstiel School of Marine & Atmospheric Science (RSMAS), University of Miami, 4600 Rickenbacker Cswy, FL 33149 Miami, USA; 2 Florida Fish and Wildlife Conservation Commission, Division of Marine Fisheries Management, University of Southern Mississippi, Gulf Coast Research Laboratory, 703 East Beach Drive, Ocean Springs, MS 39564 USA; 3 Fish and Wildlife Service 800S Guild Ave Lodi, Lodi, CA 95240 USA; 4 Florida State University, Coastal and Marine Laboratory, 3618 Coastal Highway 98, St. Teresa, FL 32358 USA; 5 Flower Garden Banks National Marine Sanctuary, 4700 Avenue U, Bldg. 216, Galveston, TX 77551 USA; 6 Present Address: Department of Biology, Geology, Physics and Inorganic Chemistry, Rey Juan Carlos University, C/Tulipán s/n, Móstoles, 28932 Spain

## Abstract

Despite the devastating impact of the lionfish (*Pterois volitans*) invasion on NW Atlantic ecosystems, little genetic information about the invasion process is available. We applied Genotyping by Sequencing techniques to identify 1,220 single nucleotide polymorphic sites (SNPs) from 162 lionfish samples collected between 2013 and 2015 from two areas chronologically identified as the first and last invaded areas in US waters: the east coast of Florida and the Gulf of Mexico. We used population genomic analyses, including phylogenetic reconstruction, Bayesian clustering, genetic distances, Discriminant Analyses of Principal Components, and coalescence simulations for detection of outlier SNPs, to understand genetic trends relevant to the lionfish’s long-term persistence. We found no significant differences in genetic structure or diversity between the two areas (F_ST_ *p-*values > 0.01, and t-test *p-*values > 0.05). In fact, our genomic analyses showed genetic homogeneity, with enough gene flow between the east coast of Florida and Gulf of Mexico to erase previous signals of genetic divergence detected between these areas, secondary spreading, and bottlenecks in the Gulf of Mexico. These findings suggest rapid genetic changes over space and time during the invasion, resulting in one panmictic population with no signs of divergence between areas due to local adaptation.

## Introduction

The Indo-Pacific lionfish (*Pterois* spp.) invasion of the northwestern Atlantic (NW Atlantic) is remarkable for its speed and magnitude^1^ and considered among the world’s more critical conservation issues during the last decade^2^. The lionfish invasion is the first recorded invasion of a marine fish species in United States Atlantic waters^3^. Lionfish are now the most abundant fish predators in many reefs within the Wider Caribbean (i.e. tropical NW Atlantic, Caribbean Sea and Gulf of Mexico)^4–6^, including those at depths of 30–350 m, with densities that, in some cases, far exceed those in their native ranges^7^. The success of these invasive predators is likely related to a combination of niche availability in the introduced area, ability to colonize a wide variety of habitats and broad thermohaline tolerance, high fecundity, wide-ranging dispersal, venomous defences, and absence of natural predators in the newly colonized environments^8^. The rapid increase in lionfish abundance in the Wider Caribbean has been linked to direct and indirect impacts on invaded ecosystems^6,7^, such as significant declines in native fish biomass due to lionfish’s predatory success on native species^7,9^ and their purported ability to displace large reef fishes, including groupers^7,9^. Lionfish are high-efficiency predators that feed primarily on post-settlement reef fishes, which they disorient by blowing water jets on them before capture^10^. Prey in the invaded range have not developed defence strategies against this predatory mechanism and so succumb easily^10^. In addition, female lionfish are capable of spawning year-round, every 2–9 days (season dependent), with an average annual output of more than 2 million eggs and several tens of generations. This tremendous output of buoyant gelatinous egg masses that drift with marine currents, provides opportunities for widespread dispersal over short time-periods^11^.

The lionfish invasion has been chronologically well documented, and potential routes of invasion and secondary spreading tracked^3,12,13^. Lionfish, likely introduced via the ornamental pet trade and aquarium releases^14–16^, was first noticed in the Western Atlantic on the east coast of Florida off Dania Beach in 1985^17^. In 1992, six lionfish were reported to have escaped from the Miami Aquarium at Key Biscayne during Hurricane Andrew^18^. By 2001, lionfish populations were well established along the US Atlantic coast, from Florida to North Carolina and Bermuda, with sporadic observations reported as far as north Rhode Island, where cold winter temperatures constraint the development of permanent populations^3^. After 2001, lionfish rapidly spread into the Bahamas and throughout Caribbean Sea, with observations of well-established populations in 2004 and 2007, respectively^3,19,20^. Following the colonization of the Caribbean, the first apparent arrival of lionfish into the Gulf of Mexico via larval transport was reported in 2009, where they quickly spread and increased in density^13,16,21^. Unfortunately, lionfish dispersion is not limited to the North Hemisphere as in 2014 recreational divers collected one adult specimen approximately 5,500 km from the Caribbean in a subtropical reef off Brazil’s southeast coast^22^.

During the last years, genetic approaches using mitochondrial DNA have been applied to investigate the lionfish invasion. Barcode analyses suggested that although two lionfish species, *Pterois volitans* (Linnaeus, 1758) and *P. miles* (Bennett, 1828), were introduced in the NW Atlantic^15,23^, *P. volitans* is the most ubiquitous species, occurring throughout the US east coast, Caribbean Sea, and the Gulf of Mexico^15,16,20,21,23,24^. Although some molecular data did not detect signs of mitochondrial introgression and/or hybridization between the two potential species^16^, the most recent morphological and molecular information revealed that *P. volitans* is a recent hybrid species between the Indian lineage of *P. miles* and a Pacific lineage encompassing *P. lunulata* and *P. russelii*^25^.

**Figure 1:**
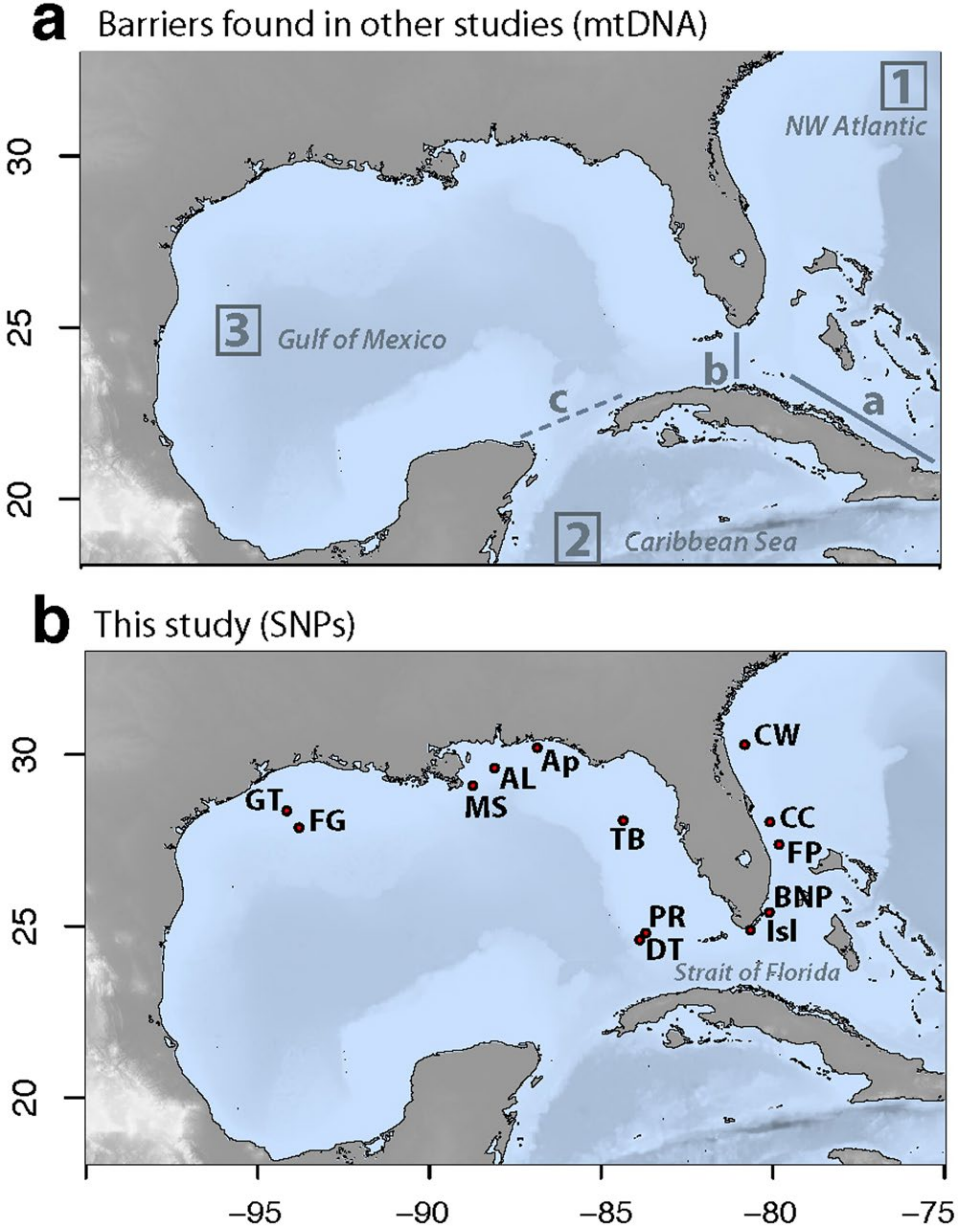
Sampling collection of lionfish. (**a**) Map showing major genetic discontinuities (**a**,**b** and **c** in blue) and genetically isolated areas (according to the temporal invasion progression as 1, 2 and 3) detected in previous studies from mitochondrial DNA of *P. volitans*^21^, and (**b**) Map of the sampling sites in this study. Maps were created for this study with the “marmap” package^73^ in R^64^.

Population genetic studies using the mitochondrial d-loop fragment of *P. volitans*, conducted across the Wider Caribbean, supported the chronological records of the invasion and confirmed the colonization routes followed by the species^15,16,20,21,23,26,27^. While these studies discarded the hypothesis of multiple introductions from native sources during the invasion process, they revealed successive bottlenecks over the course of the invasion. Across the Wider Caribbean, only nine d-loop haplotypes were identified among the total 1,248 samples sequenced^16,20,21,26,27^, a number that contrasts with the 37 different haplotypes sequenced in the native range at the Indo-Pacific in only 70 specimens^20^. D-loop analyses showed that the first bottleneck occurred from the native range to the NW Atlantic, although the NW Atlantic area (east coast of the US), identified as the entrance point of lionfish, displayed the highest nucleotide and haplotype diversity and number of haplotypes within the Wider Caribbean, with the nine different haplotypes^16^. The second bottleneck occurred within the invasive range when of the nine haplotypes found at the NW Atlantic only four secondarily spread to the Caribbean Sea. Finally, three of the four haplotypes found in the Caribbean Sea invaded the Gulf of Mexico^16,20,21,26,27^. Hence, the lionfish invasion initially contrasts with the perception that avoiding genetic bottlenecks by the influx of genetic diversity through repeated introductions from the native range increases the invasion success and posterior spread within the invasive area^28,29^, as observed in a number marine invasions fuelled by multiple introductions from different native sources^30,31^. The study by Johnson and co-authors (2016), which pooled together all d-loop sequences obtained from the Wider Caribbean between 2007 and 2013, also detected significant differences in genetic structure among the three mentioned areas: NW Atlantic, Caribbean Sea, and the Gulf of Mexico (See Fig. 1), but no significant differences within them^21^, despite the genetic divergence noticed among some populations of the Caribbean Sea in a previous study^27^. Additionally, the sharp genetic discontinuity between the NW Atlantic and the Gulf of Mexico suggested no direct gene flow across the Strait of Florida^21^.

Despite the important insights revealed by the d-loop, using only one mitochondrial marker may prevent detection of fine-scale differentiation and connectivity within lionfish’s invasive range^16^, and hence nuclear loci are required to provide a complete picture of the current genetic status of this invasion. In this study, we focus our attention on *P. volitans*, referred to as “lionfish” hereafter. We analyse two areas chronologically identified as the first and last invaded areas within the Wider Caribbean: the NW Atlantic (east coast of Florida) and the northern Gulf of Mexico, respectively. These two marine areas displayed highly significant differences in genetic diversity, structure and absence of connectivity across the Florida Strait for the d-loop marker in a previous study^21^.

We here apply Genotyping by Sequencing (GBS) techniques to identify over a thousand nuclear and independent single nucleotide polymorphic sites (SNPs) from samples collected over a short time. Our general aim is to understand the invasion progression by determining fine-scale population genomics between the NW Atlantic (east coast of Florida) and the northern Gulf of Mexico, and potential connectivity between them, knowing that the spatial genetic structure, temporal genetic trend, and levels of connectivity among areas is relevant to predict the potential long-term persistence of an invader^29^ and to design management strategies for its control.

## Results

From the Genotyping by Sequencing (GBS) library of 229 lionfish samples, a total of 404,254 sequence tags were retained in TASSEL^32^. Filtering for individuals with at least 75% of the called loci and loci that were present in at least 84% of individuals yielded a total of 1,654 SNPs and 162 individuals from the NW Atlantic and Gulf of Mexico (see Tables 1 and 2, and Fig. 1). From this dataset, 434 SNPs were removed: 61 SNPs that showed significant linkage disequilibrium (r^2^ > 0.2999. FDR *p*-value < 0.01) and 373 SNPs with significantly greater observed than expected heterozygosity (Hardy Weinberg Equilibrium- HWE. *p-*value < 0.01) (see Supplementary Material FS1), leaving a final dataset of 1,220 SNPs in 162 specimens covered by 2,322,797 reads (mean: 11.75 reads per locus and sample).

**Table 1:** General information of lionfish samples. Sampling site, major marine area, site code, number of individuals genotyped (Ng), year of collection (Date), geographical coordinates (Latitude and Longitude), collection depth (in meters), and preservation method.

Site	Area	Code	Ng	Date	Latitude	Longitude	Depth	Preservation
North East. FL	NW Atlantic	CW	10	2015	30.29	−80.82	no data	Ethanol
Cape Canaveral. FL	NW Atlantic	CC	6	2015	28.04	−80.09	27	Ethanol
Biscayne National Park. FL	NW Atlantic	BNP	38	2015	25.4	−80.1	4 to 36	Chaos
Ft. Pierce. FL	NW Atlantic	FP	5	2015	27.38	−79.82	24	Ethanol
Islamorada. FL	NW Atlantic	Isl	30	2015	24.88	−80.65	no data	Chaos
Dry Tortugas. FL	Florida Keys	DT	19	2013	24.59	−83.88	62	Ethanol
Pulley Ridge	Florida Keys	PR	26	2013	24.8	−83.70	62	Ethanol
Tampa. FL	Gulf of Mexico	TB	19	2014	28.08	−84.36	34	Ethanol
Apalachicola. FL	Gulf of Mexico	Ap	19	2014	30.2	86.86	35	Ethanol
Alabama Shelf. AL	Gulf of Mexico	AL	20	2014	29.61	−88.101	39	Ethanol
Mississippi Delta. LA	Gulf of Mexico	MS	10	2013	29.09	−88.734	44	Ethanol
Flower Garden Banks. TX	Gulf of Mexico	FG	10	2015	27.87	−93.80	24	Frozen + ethanol
Galveston. TX	Gulf of Mexico	GT	17	2014	28.36	−94.157	27	Ethanol

**Table 2:** Main genetic descriptors for all 1,220 SNPs.

Code	Nf	mean n° alleles	Ho	He	F_IS_	HWE
CW	7	1.85 ± 0.001	0.268 ± 0.006	0.289 ± 0.005	0.054 ± 0.013	0.001
CC	1	0.87 ± 0.017	0.121 ± 0.009	0.061 ± 0.005	—	—
BNP	36	1.99 ± 0.002	0.358 ± 0.006	0.337 ± 0.004	−0.027 ± 0.001	1.000
FP	4	1.76 ± 0.012	0.230 ± 0.008	0.280 ± 0.005	−0.080 ± 0.018	1.000
Isl	29	1.99 ± 0.002	0.359 ± 0.006	0.337 ± 0.004	−0.033 ± 0.001	1.000
DT	6	1.74 ± 0.013	0.186 ± 0.006	0.256 ± 0.005	0.216 ± 0.015	0.001
PR	20	1.98 ± 0.004	0.294 ± 0.005	0.310 ± 0.004	0.057 ± 0.01	0.001
TB	11	1.90 ± 0.009	0.225 ± 0.004	0.288 ± 0.005	0.193 ± 0.013	0.001
Ap	10	1.88 ± 0.009	0.237 ± 0.005	0.289 ± 0.004	0.151 ± 0.012	0.001
AL	20	1.98 ± 0.004	0.309 ± 0.006	0.320 ± 0.004	0.053 ± 0.011	0.001
MS	1	0.84 ± 0.016	0.099 ± 0.009	0.050 ± 0.004	—	—
FG	4	1.58 ± 0.015	0.191 ± 0.007	0.220 ± 0.006	0.081 ± 0.017	0.001
GT	13	1.93 ± 0.007	0.256 ± 0.005	0.298 ± 0.004	0.120 ± 0.012	0.001

Population code, number of individuals retained for analyses after filtering (Nf), mean number of alleles, observed and expected heterozygosity (Ho and He, respectively), Fixation index (F_IS_) and *p*-values of Hardy Weinberg Equilibrium, Mean number of alleles, Ho, He and F_IS_ are presented with standard errors.

### Detection of SNP outliers

**Figure 2:**
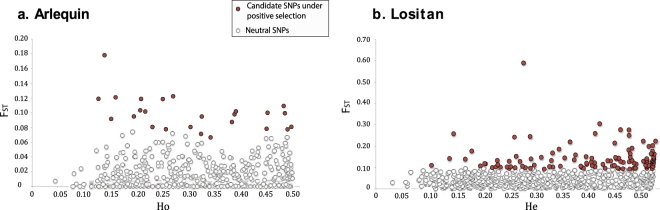
Detection of outlier SNPs. (**a**) Using coalescent simulations with Arlequin: F_ST_ and observed heterozygosity (Ho), and (**b**) Coalescent simulations using Lositan: F_ST_ and expected heterozygosity (He). Neutral SNPs are plotted as white circles and candidate SNPs under positive selection are represented as red circles. Candidate SNPs under balancing selection are not represented in this graph.

From the dataset of 1,220 SNPs, 23 outlier SNPs were identified as candidate markers under positive selection with Arlequin after FDR correction (24 SNPs were identified when the uncorrected *p*-value ≤ 0.01 was applied). No marker was identified to be under balancing selection from Arlequin (see Fig. 2). Lositan identified a total of 213 outlier SNPs, 73 of them candidates under positive selection (Fig. 2) and 140 candidates under balancing selection. Among all outlier SNPs, only 13 were found in common between both methods. We consider that only these 13 outlier SNPs had strong statistical support to be considered as potentially under positive selection. The remaining 1,207 SNPs were assumed to be neutral, although their neutrality could not be directly proven.

### Lack of divergence between the NW Atlantic and the northern Gulf of Mexico

The main genetic descriptors obtained for all 1,220 lionfish SNPs are listed in Table 2. Most populations showed lower values of observed (Ho) than expected heterozygosity (He), which translated into positive values of the fixation index F_IS_, and significant deviation from HWE (Table 2). Genetic diversity values varied among populations: populations with only few individuals had the lowest diversity. For those populations with 10 or fewer individuals, the mean number of alleles per locus was lower than the potential maximum of 2 (see the curve of accumulative number of alleles related to sample size in Supplementary Material FS2). Our analyses did not detect significant differences in genetic diversity (assessed as Ho and He) between the NW Atlantic and the Gulf of Mexico (t-tests: *t* = 0.89 and 0.42, *p-*values = 0.39 and 0.68, respectively).

The Maximum Likelihood (ML) tree reconstructed from 1,220 SNPs of 162 specimens from 13 locations did not show geographical clustering of specimens related to the sampling site and/or geographical area where they were collected (Supplementary Material FS3) and displayed very low bootstrap value support on most nodes.

**Figure 3:**
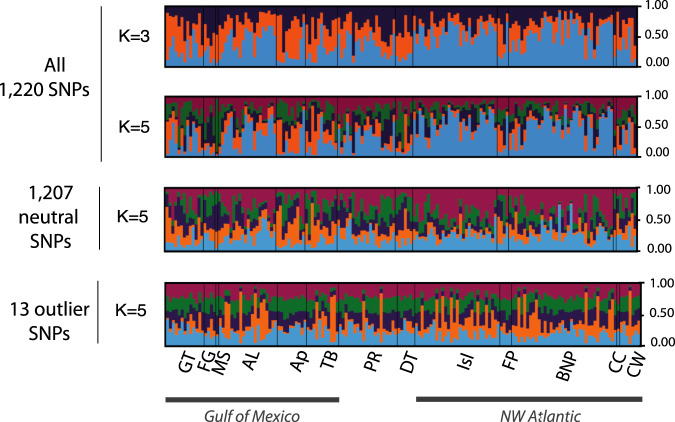
Structure barplot outputs. Posterior probabilities of individual assignment of the most probable number of clusters (K = 3 and 5; different clusters are represented by different colours) for all 1,220 SNPs. Barplots from 1,207 neutral SNPs and 13 candidate SNPs under selection (outliers) are also presented. For neutral and outlier SNPs the graph of K = 5 is shown for comparison with the whole dataset.

In the Bayesian clustering analysis performed in Structure for all 1,220 SNPs, the optimal numbers of homogeneous genetic clusters (K) for the whole data set^33^ were three and five (K = 3 and K = 5) according to the ad hoc statistic ΔK (see values of Delta K- ΔK- in Supplementary Material FS4), but the Log likelihood for K (LK) did not significantly increased from 1 to 5 suggesting lack of spatial genetic clustering (see LK in Supplementary Material FS4). The individual-based cluster memberships from K = 2 to K = 8 (see Supplementary Material FS4) showed no spatial genetic heterogeneity among sampling sites and mixed membership of individuals to all clusters, supporting the hypothesis of panmixia across the Florida Strait (see Fig. 3 for K = 3 and K = 5, and Supplementary Material FS4 for all K values). Only slight differences in terms of a higher probability of one specific cluster (the blue cluster) could be observed in two sites located at south Florida, BNP and Isl (Fig. 3). Genetic admixture across the invasive range was also observed when 1,207 neutral SNPs and 13 outlier SNPs were separately analysed. In Fig. 3, we compare Structure results for K = 5 from the three different datasets.

The Analyses of Molecular Variance (AMOVA) for all 1,220 SNPs, 1,207 neutral SNPs, and 13 outlier SNPs also did not detect genetic differentiation associated with the NW Atlantic and Gulf of Mexico (“Among groups”), regardless of whether the two populations from the Florida Keys (Dry Tortugas- DT and Pulley Ridge- PR) were pooled or removed from the analyses, and most genetic variation was retained within individuals (Table 3). No differences were observed from AMOVA results between the datasets including all 1,220 SNPs and only 1,207 neutral SNPs (see Table 3 and Supplementary Material TS1). Nevertheless, from the 13 outlier SNPs, we detected significant differences at two additional variance components: between populations within the NW Atlantic and Gulf of Mexico, and among individuals within populations, but still most genetic variation was retained within individuals (Table 3). F_ST_ statistics gave us more details of the pairwise differences between populations within the NW Atlantic and the Gulf of Mexico. The F_ST_ statistics were in general very low, and only two pairwise comparisons were significant (between AL and BNP, and Isl) after FDR correction of the *p*-values when all 1,220 SNPs (Table 4) or only 1,207 neutral SNPs (data not shown) were included in the analyses. F_ST_ distances from the 13 outlier SNPs revealed significant genetic differentiation between other sites: Ap and FG seemed to be the most genetically divergent sites (see Table 4), but this genetic divergence was not fully supported by Bayesian clustering analyses (see previous results from Structure) or discriminant analyses of principal components (see explanation below).

**Table 3:** Results of analyses of molecular variance (AMOVAs) of lionfish from all 1,220 SNPs and 13 candidate SNPs under selection (outliers).

Source of variation	d.f	% Var.	Fixation index	*p-*value	% Var.	Fixation index	*p-*value
		All 1,220 SNPs			13 outlier SNPs		
***PR and DT within NW Atlantic***							
Among groups	1	0	−0.001	0.99	0	−0.012	0.58
Among populations within groups	11	0	−0.006	0.99	5.7	0.056	0.00
Within populations	149	0	−0.036	1	27.3	0.286	0.00
Within individuals	162	100	−0.044	0.85	68.2	0.318	0.00
***PR and DT within Gulf of Mexico***							
Among groups	1	0	−0.002	0.90	0	−0.012	0.58
Among populations within groups	11	0	−0.006	1	5.7	0.056	0.00
Within populations	149	0	−0.037	0.98	27.3	0.286	0.00
Within individuals	162	100	−0.449	0.95	68.2	0.318	0.00
***Without PR and DT***							
Among groups	1	0	−0.002	0.87	0	−0.016	0.52
Among populations within groups	11	0	−0.005	0.99	7.04	0.069	0.00
Within populations	125	0	−0.045	0.98	28.3	0.299	0.00
Within individuals	136	100	−0.053	0.99	68.2	0.338	0.00

Populations’ grouping (Source of variation), degrees of freedom (d.f.), percentage of variation (% Var.), fixation indexes, and *p*-values.

**Table 4:** Lionfish F_ST_ values between sampling sites.

	CW	BNP	FP	Isl	DT	PR	TB	Ap	AL	FG	GT
**CW**	—	0.05904	0.12398	0.07693	0.08734	0.08357	0.09997	0.12432	0.02388	0.26866	0.11102
**BNP**	−0.00585	—	0.13273	0.01039	0.05440	0.01263	0.04417	0.08623*	0.01792	0.08177	0.05091
**FP**	−0.0091	−0.01163	—	0.15015	0.11214	0.13219	0.12923	0.13048	0.12197	0.28386	0.10115
**Isl**	−0.00569	−0.00205	−0.00986	—	0.01259	0.00150	0.00448	0.08536*	0.00696	0.13068*	0.03878
**DT**	−0.01861	−0.03608	−0.01768	−0.04071	—	−0.04126	0.05904	0.04868	0.01374	0.30744	−0.03108
**PR**	−0.00091	−0.00059	−0.00483	−0.00267	−0.02645	—	0.04344	0.03418	0.00650	0.16721	−0.00889
**TB**	−0.00174	−0.01366	0.00119	−0.01649	−0.00057	−0.00173	—	0.11251	0.01298	0.26521*	0.01745
**Ap**	−0.00228	−0.01181	−0.0075	−0.01483	−0.00275	−0.00333	0.01082	—	0.09401*	0.26914*	0.02245
**AL**	−0.00374	0.00136*	−0.00754	0.00211*	−0.02251	0.00291	−0.00487	−0.0038	—	0.20537*	0.01672
**FG**	−0.00706	−0.03463	−0.00706	−0.03926	0.01363	−0.023	0.01023	0.01088	−0.02301	—	0.24847*
**GT**	0.00236	−0.00695	−0.00131	−0.00917	−0.01311	−0.00199	−0.00225	−0.00287	−0.00034	−0.00234	—

F_ST_ values from all 1,220 SNPs below the diagonal, and 13 candidate SNPs under selection (outliers) above the diagonal. *Significant after false discovery rate (FDR) correction set at *p* ≤ 0.01.

**Figure 4:**
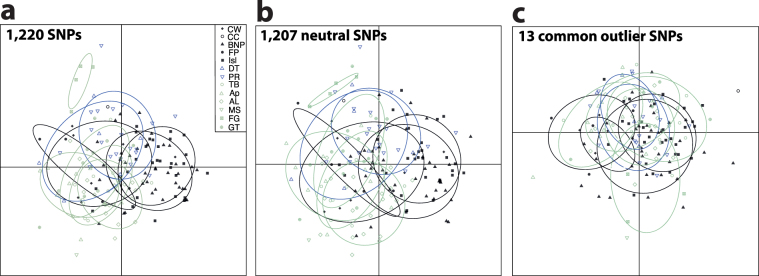
DAPC results. The DAPC graphs represent results from three different analyses: (**a**) from all 1,220 SNPs, (**b**) 1,207 neutral SNPs, and (**c**) 13 candidate SNPs under selection (outliers). In the DAPC graph points represent different individuals, point patterns different sampling sites, and colours different marine areas (grey = NW Atlantic, blue = Dry Tortugas and Pulley Ridge, and light green = northern Gulf of Mexico).

The discriminant analyses of principal components (DAPC) also showed a general pattern of low genetic differentiation, as that observed from previous analyses. According to the Bayesian Information Criterion (BIC) that compares different DAPC clustering solutions, two clusters were the optimal number to describe our data. The DAPC plot, including all sampling sites and all 1,220 SNPs, showed no clear separation of populations or clusters between the NW Atlantic and Gulf of Mexico (Fig. 4a). Only FG seemed lightly isolated from all the other sampling sites. This pattern was maintained when only 1,207 neutral SNPs were included in the DAPC analysis (Fig. 4b). When 13 outlier SNPs were separately analysed, the divergence between FG and all the other populations decreased (Fig. 4c).

## Discussion

Our genomic data of 1,220 SNPs from 162 *P. volitans* specimens across the NW Atlantic and the northern Gulf of Mexico represents the first study using nuclear loci to explore the genetic structure of this invasive predator and is among the few studies applying genome-wide scanning, based on Next Generation Sequencing technologies, to investigate population structure of a marine invader (see a review in ref.^34^, and examples in refs^35,36^). Although 18 nuclear microsatellite loci were isolated for *P. volitans* and *P. miles* a few years ago^37^, to our knowledge, those markers have not yet been used for population analyses.

Our fine-scale population genomic analyses of lionfish demonstrate lack of a current genetic break between the first and the last invaded areas in US waters: the NW Atlantic and the Gulf of Mexico, respectively. The Bayesian clustering analysis and DAPC showed different clustering solutions due to a lack of clear genetic differentiation across the whole analysed area. From all 1,220 SNPs and 1,207 neutral SNPs, we only noticed significant genetic differences between three sites based on F_ST_ distances; the 13 outlier SNPs, FG and Ap showed significant differences with five additional sites, but that divergence was not mirrored by other analyses (e.g. Structure and DAPC). Only FG seemed to be genetically divergent for most analyses and databases. However, this location only includes four individuals, a sample size that cannot be considered representative of the genetic diversity and structure of this location, as demonstrated by the low number of accumulated alleles within these four individuals (see Table 2). Hence, independently of the database used (all 1,220 SNPs, 1,207 neutral SNPs, or 13 outlier SNPs), results show a picture of general genetic homogeneity with enough gene flow between the NW Atlantic and the Gulf of Mexico to erase signals of secondary spreading detected from d-loop analyses in previous years and studies^23^. Whether current gene flow occurs directly across the Florida Strait or indirectly throughout the Caribbean Sea cannot be assessed by the presented data, and further analyses including samples from the Caribbean Sea are necessary to investigate connectivity routes.

Our findings, therefore, contrast with the discontinuity between the NW Atlantic and the Gulf of Mexico and the lower genetic diversity of the Gulf of Mexico observed in previous studies based on d-loop data^16,20,21,27^. Different mitochondrial and nuclear DNA patterns (mito-nuclear discordance), such as those noticed here for lionfish, are becoming more commonly reported as the number of nuclear multilocus datasets increases (see examples in refs^38,39^) and can be explained by several non-exclusive causes^40^. Demographic asymmetry due to sex-biased dispersal can cause mito-nuclear discordance in motile animals, including marine fish species^40,41^. Although recent findings suggest that lionfish adults move more than initially thought^42^, they are not migratory, and buoyant eggs are the dispersal stage^11^, so different migratory behaviour between males and females cannot explain the pattern we found, and other hypotheses should be considered, including temporal genetic shifts and selection.

A plausible cause of discordance between lionfish studies using different markers is temporal changes in the genetic structure over the invasion process. Population genetics theory anticipates fast genetic changes in introduced populations characterized by bottlenecks, founder effects, strong genetic drift, and new selective pressures in the introduced environments^43–45^. Despite the importance of temporal genetic trends for the invasion dynamics, this point is overlooked in most studies of marine invaders, and it is assumed that genetic diversity remains stable over time^34^. The few studies investigating temporal trends of genetic structure in introduced marine species showed variable outcomes^46–51^. Whereas some invasive ascidians suffering massive seasonal die-off events maintained stable levels of genetic diversity and homogeneous structure over time due to the re-establishment of populations from the survivors or recolonization from nearby sites^48,51^, other introduced species exhibited changes in genetic architecture over short time periods. For instance, in the introduced colonial ascidian, *Perophora japonica*, a genetically isolated population from Plymouth (South England) displayed a linear reduction in mitochondrial genetic diversity and large haplotype frequency changes over a 9 year-monitoring period, due to either genetic drift and/or selection^46^. Rapid allele frequencies changes over time, high heterozygous deficiency, and inbreeding were also detected in isolated populations of the colonial ascidian *Botryllus* along the coast of Israel^49^, but genetic isolation was not always associated with genetic diversity loss in this species. Invasive *Botryllus* populations along the Californian coast, isolated from other genetic sources and highly influenced by genetic drift and selection, maintained stable levels of genetic diversity thanks to high mutation rates generating a complex pattern of allele gains and losses^47^. Nevertheless, marine invasive populations are, in many cases, characterised by high levels of genetic diversity due to multiple introductions from genetically distinct sources^28^. An outstanding example of multiple cryptic introductions and genetic admixture within the invaded range, which might be related to the high invasion success, is that of the European green crab, currently one of the most important aquatic invaders established across all temperate shores around the world^30,36^.

In lionfish, d-loop data revealed strong bottlenecks and scientists discarded the idea of multiple introductions into the NW Atlantic from the native range^16,20^, which would result in a small initial effective population size. Moreover, changes in genetic structure are expected to occur faster in mitochondrial than nuclear DNA because mitochondrial DNA, a haploid, maternally inherited molecule, has an effective population size of one-quarter that of nuclear DNA and therefore is more sensitive to diversity changes associated with genetic drift. For this reason, the comparison of the NW Atlantic d-loop data (collected between 2007 and 2009) and the Gulf of Mexico (collected between 2011 and 2013)^16,20,21,27^ should be taken with caution since it assumes that NW Atlantic populations remained static over a six-year period with no changes in haplotype frequencies due simply to genetic drift. This assumption of temporal stability might obscure the most recent pattern of genetic diversity in lionfish. In this sense, the analyses presented here, based on 1,220 nuclear SNPs from samples collected during a brief period of 20 months seems to be a more reliable way to determine lionfish’s current genetic structure.

Besides neutral temporal trends, differential selection can also promote discrepancy patterns between mitochondrial and nuclear DNA. Mitochondrial selection under divergent environmental conditions plays an important role for the distribution of mitochondrial variants in other marine fish species^52,53^. Different environmental pressures between the NW Atlantic and the Gulf of Mexico could also have favoured some lionfish mitochondrial haplotypes over others, thus shaping the spatial distribution of haplotypes during the invasion process. However, the sampling scheme used in our study and the lack of new mitochondrial sequences do not allow mitochondrial selection and/or adaptation hypotheses to be tested. None of the 1,220 tags containing the SNPs were identified as mitochondrial fragments, and the different analyses performed did not reveal evidence of local adaptation and/or nuclear selection across the lionfish’s invasive range. In some marine species with long-dispersal potential, outlier SNPs unravelled significantly finer genetic structure than neutral markers, suggesting the existence of local adaptation^36,54–57^. For example, in the European hake, Atlantic and Mediterranean populations showed sharper divergence in analyses using outlier SNPs than neutral SNPs^54^, a pattern of higher resolution that was also found in other fish species at small geographical scales of a few hundred kilometres when analysing outlier SNPs^55,56^. In some marine invaders, local adaptation also seemed to play an important role in shaping populations’ genetic structure, showing either latitudinal clines in outlier allele frequencies^36^ or significant correlation with environmental variables such as salinity and water temperature^57^. Nevertheless, in lionfish, differential selection between the NW Atlantic and the Gulf of Mexico, and mito-nuclear interactions remains as an open question because although we did not find strong evidence of local adaptation, 1,220 SNPs still represent a small proportion of the species’ genome, and selection on non-explored genomic areas could be possible.

The SNP data here presented yield valuable information of the genetic trend in the lionfish invasion, which could potentially be affected by genetic changes over time and across space, although other hypotheses such as different selective pressures between mitochondrial and nuclear DNA cannot be completely discarded. Additionally, we perceive some limitations in our study that should be taken in consideration for further investigations. For instance, we noticed that population analyses based on SNPs should include: sizes over 10 individuals to retain the potential maximum genetic diversity within populations, representative populations from the native range to shed light on the current impact of bottlenecks in genetic diversity across the whole invasive range, and populations from the Caribbean Sea to clarify the most important connectivity routes within the invaded area.

As demonstrated by previous publications based on mitochondrial DNA, which detected strong bottlenecks during the first introduction steps and invasion progression^16,20,21,27,28^, the lionfish invasion is an example of how reductions in genetic diversity do not necessarily compromise population establishment and spreading^16,20,21,27,28^ and points out the importance of primary (pre-border) and secondary (post-border) introductions, e.g., secondary introductions to the Caribbean Sea and later to the Gulf of Mexico^16,20,21,27^. The potential of lionfish to overcome these initial steps of the invasion with low genetic diversity at the mitochondrial DNA^16,20,21,27^, and to homogenize nuclear genetic structure across the invaded area (as shown in this study with SNPs), should be considered when developing theoretical models on the expected geographical spreading of this invasion^58^ and implementing appropriate strategies for its management and control.

Finally, the lionfish invasion to the Wider Caribbean can be used as a lesson to anticipate the genetic trend and potential impacts of *P. miles* invasion in the Mediterranean Sea. As *P. volitans* across the Wider Caribbean, *P. miles* has quickly colonized wide areas of the eastern Mediterranean^59^, which adds an additionally threaten in a small sea that is at the same time a hotspot of marine biodiversity and one of the world’s most impacted seas.

## Methods

### Sampling collection

*P. volitans* samples were collected over a 20 month period, between June 2013 and February 2015, from thirteen locations along Florida’s eastern coast (NW Atlantic and Florida Keys) and the northern Gulf of Mexico, at depths between 4 and 62 meters. Sampling sites and number of individuals genotyped are detailed in Table 1 and Fig. 1. Collections were often opportunistic by SCUBA divers, so collection depths could not always be recorded. Fin or gill clips were obtained from the collected specimens and preserved in absolute ethanol, frozen at −20 °C or stored in 320 µl of chaotropic buffer (4.5 M guanadinium thiocynate, 2% N-lauroylsarcosine, 50 mM EDTA, 25 mM Tris-HCL pH 7.5, 0.2% antifoam, 0.1 M β-mercaptoethanol) (see Table 1).

#### Ethics Statement

No endangered or protected species were involved in this study. Lionfish were sampled opportunistically by the authors from lionfish derbies or state and federal collections (as stated below); only dead lionfish were obtained. Lionfish were collected by a number of organizations in areas open to fishing with a spear or permitted by methods utilized. These fish were collected as a result of other activities such as tournaments, commercial harvest, and general fisheries surveys, and were sampled opportunistically for this study. No permits were required to collect lionfish beyond a state saltwater fishing license, which was in possession of divers at each collection. In the case of lionfish collected from offshore Florida, no fishing license is required. The University of Miami Institutional Animal Care and Use Committee (IACUC) did not require a protocol for this study since only dead specimens were donated to the University. State and Federal government organizations, although exempt from IACUC requirements, follow best practices to minimize pain and suffering of specimens. These are approved Institutional Animal Care and Use Committee protocols via the American Veterinary Medical Association Guidelines for the Euthanasia of Animals and the American Society of Ichthyologists and Herpetologists Guidelines for Use of Fish in Research.

### Library construction and SNP isolation

Genomic DNA was extracted from tissue clips using silica columns. DNA quality was assessed via agarose gel electrophoresis, and DNA concentrations were quantified using Biotium AccuBlue^TM^ Broad Range dsDNA Quantitative Solution according to the manufacturer’s instructions. After quantification, 100 ng of DNA from each sample was dried down in 96-well plates in a SpeedVac concentrator. Samples were then rehydrated overnight with 5 µl of ultrapure milliQ water before further processing.

Genotyping by Sequencing libraries were constructed using the restriction enzyme *ApeKI*. A total of 50 ng of genomic DNA per sample was digested at 75 °C for 2 hours. Unique barcoded adapters were used for library construction as described in^60^. A total of 229 DNA samples were pooled together and fragments approximately 300 bp in length were selected with magnetic beads. Primers complementary to the adapters were then used for library amplification^60^. Before sequencing, library quality was checked in an Agilent 2100 Bioanalyzer. The GBS library including the 229 individuals was sequenced in two lanes of an Illumina Hi Seq. 2500 using 75 bp single end reads at Elim Biopharmaceuticals, Inc. Hayward, CA.

The UNEAK GBS analysis pipeline in TASSEL^32^ for species without a reference genome was used to call SNPs using Bowtie^61^. The software identifies SNPs found on single non-overlapping “tags” (64 bp sequences) initiated at the restriction sites. Only SNPs that had a minimum of five reads across all samples were retained to reduce the impact of sequencing errors. Loci with significant linkage disequilibrium (D’ *p*-value False Discovery Rate correction-FDR- adjusted to 0.01) identified in TASSEL and those with significantly greater observed than expected heterozygosity (*p-value* < 0.01) were removed from the database before performing further analyses. SNPs were then filtered to select individuals with at least 75% of the called loci and loci that were present in at least 84% of individuals. Maximum heterozygosity during filtering was set at 0.5 to avoid excess of heterozygotes due to sequencing errors. All sequences containing selected SNPs were blasted (e-value < 10^−5^) against the mitochondrial DNA of *Salmo salar* and the Genbank database to identify mitochondrial fragments.

### Data availability

The HapMap file including the whole dataset of SNPs here analysed, a coverage file and the genepop file including allele frequencies have been deposited in PANGAEA (10.1594/PANGAEA.886118).

### Detection of outlier SNPs

Two different software programs, Arlequin^62^ and Lositan^63^, were used to identify non-neutral SNPs, as candidate markers under selection, based on an F_ST_-outlier detection method and coalescence simulations. Arlequin uses simulations based on observed heterozygosity (Ho) to create a null distribution of F_ST_ values and associated *p*-values for each locus. We performed a total of 20,000 simulations, with 100 demes, under a finite island model. This model was chosen due to the general lack of genetic structure (see Results). FDR correction of the *p*-values was applied to detect significant outliers; we also considered a more conservative approach with significance at *p* < 0.01 since strong corrections can increase type II error thereby assuming neutrality in SNPs that are not neutral^55^ (although both approaches showed similar results). Lositan, on the other hand, creates a distribution based on the relation between F_ST_ values and expected heterozygosity (He). We performed a first run using all loci to estimate mean F_ST_ values with 20,000 simulations, 99% confidence interval, infinite alleles mutation model and false discovery rate of 0.1%. After the first run, loci in the confidence interval were removed, and “neutral” F_ST_ values were recalculated. A third run was finally performed using all loci, and the neutral F_ST_ values previously calculated were implemented to detect outliers. Finally, outliers recovered from both software programs, Arlequin and Lositan, were considered as candidate SNPs under selection.

### Genetic structure analyses

General descriptors of genetic diversity as mean number of alleles, observed heterozygosity (Ho), expected heterozygosity (He), fixation index F_IS_, and the Hardy Weinberg Equilibrium were calculated for all markers per population using Arlequin 3.5.1.2^62^ and the “adegenet” package in R^64^.

A Maximum Likelihood (ML) tree, including all genotypes obtained, was reconstructed in RAxML with a GTR+ G model and 100 rapid bootstrap replicates^65^ to explore potential clustering of individuals related to different geographical areas and/or sampling sites. The ML tree was then visualized and edited in Figtree 1.4.0 (http://tree.bio.ed.ac.uk/software/figtree/).

A Bayesian clustering analysis, performed with the software Structure 2.3.4^66^, was used to investigate the optimal number of major homogeneous genetic clusters (K) found within our datasets under the null hypothesis of genetic homogeneity. Because Bayesian analysis can be computationally very intense and long, an initial fast run was performed with a K from 1 to 13 with five independent replicates, 20,000 Markov chain Monte Carlo (MCMC) per replicate, and a 2,000 burn-in period to get a general idea about the maximum number of clusters expected. Then, a definitive run was performed with K from 1 to 8 with five independent replicates, 100,000 MCMC per replicate, and a 10,000 burn-in period. We used an “admixture model” and correlated gene frequencies as implemented in Structure. The five independent runs were averaged using the clumpak server^67^ (http://clumpak.tau.ac.il). The K value was determined by comparing the rate of change in the likelihood of K, using the ad hoc statistic ΔK in Structure Harvester 0.6.94^68^.

Analyses of Molecular Variance (AMOVA), based on allele frequencies, were performed to specifically explore the potential genetic break between the two genetically different regions previously identified from mitochondrial DNA, the NW Atlantic and the Gulf of Mexico. The locations of PR and DT, at the Florida Strait, are rich mesophotic reefs and part of the Florida Keys reef complex but far inside the Gulf of Mexico. Since the genetic break between the NW Atlantic and the Gulf of Mexico shifts at different points of the Florida Strait depending on the species^69,70^, we could not *a priori* assign these two sites (PR and DT) to one or the other area. Therefore, we performed three different AMOVA analyses: the first analysis included PR and DT within the NW Atlantic pool, the second included them within the Gulf of Mexico pool, and the third one excluded these two sites from the analysis. After testing differences between major marine areas, pairwise F_ST_ distances based on allele frequencies between all sampling sites were calculated with the same software. The significance of AMOVA and F_ST_ values was assessed after 50,000 non-parametric permutations of individuals among populations and/or populations between geographical areas and under the null hypothesis of genetic homogeneity. FDR correction of these *p*-values was applied for F_ST_ multiple testing^71^.

Significant difference in genetic diversity (Ho and He) between the two marine regions, the NW Atlantic and Florida Keys (CW, CC, FP, BNP, Isl, PR and DT, see Results section), and the Gulf of Mexico (TB, Ap, AL, MS, FG, GT) was evaluated with a t-test.

Additionally, discriminant analyses of principal components (DAPC)^72^ were computed for the complete dataset. DAPC does not assume any underlying population genetic model and is not as affected by Hardy Weinberg disequilibrium as other methods based on genetic distances (e.g. F_ST_ and AMOVA) and Bayesian clustering analyses. We used collection sites as populations with the “adegenet” package in R^64^. DAPC extracts multivariate information from genetic datasets by first performing a principal component analysis (PCA) on predefined groups (collection sites in this case) and then using the PCA factors as variables for a discriminant analysis (DA), which seeks to maximize the inter-site component of variation. Thus. DAPC allows the visual identification of genetic clusters and can outperform more computer-intensive approaches, such as Structure, in detecting genetic structure^72^. Since the number of principal components (PCs) retained may have large impact on the DAPC output, the optimal number of PCs to be retained was first explored by the cross-validation method implemented by this package.

To understand whether selection and/or local adaptation within the lionfish’s invasive range is an important driver of the genetic structure, the searching strategy explained before for the Bayesian clustering analysis, AMOVA, F_ST_ and DAPC was comparatively applied to three different SNP datasets: for all isolated SNPs, for neutral SNPs, and for candidate SNPs under selection (outliers).

## Electronic supplementary material

Supplementary Information
