# Supplementary material for: Genetic homogeneity of the invasive lionfish across the Northwestern Atlantic and the Gulf of Mexico based on Single Nucleotide Polymorphisms
Source: Sci Rep. 2018 Mar 22;8:5062. doi: 10.1038/s41598-018-23339-w (PMC5864727; doi:10.1038/s41598-018-23339-w)
Supplement: Supplementary file 1 — Supplementary Information [file 41598_2018_23339_MOESM1_ESM.pdf]

SUPPLEMENTARY MATERIAL

Genetic homogeneity of the invasive lionfish across the Northwestern Atlantic and the Gulf of Mexico based on Single Nucleotide Polymorphisms

R. Pérez-Portela[1 *], A. Bumford[1^], B. Coffman[1^], S. Wedelich[1^], M. Davenport[1], A. Fogg[2], M.K. Swenarton[3], F. Coleman[4], M.A. Johnston[5], D.L. Crawford[1], and M.F. Oleksiak[1]

Figure FS1. Heterozygosity graph. Graph of heterozygosity observed (Ho) *versus* expected (He) for 1,220 *P. volitans* SNPs included in this study. SNPs displaying larger and significant values of observed *versus* expected heterozygosity were removed from the database before further analyses.

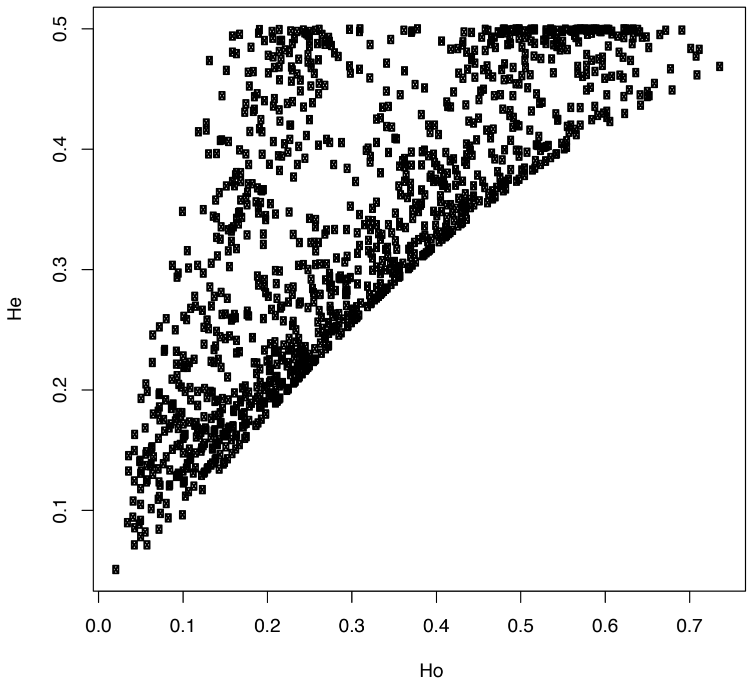

Figure FS2. Number of alleles from SNPs. Graph of the cumulative number of alleles from 1,220 SNPs of 162 *P. volitans* specimens related to population sample size.

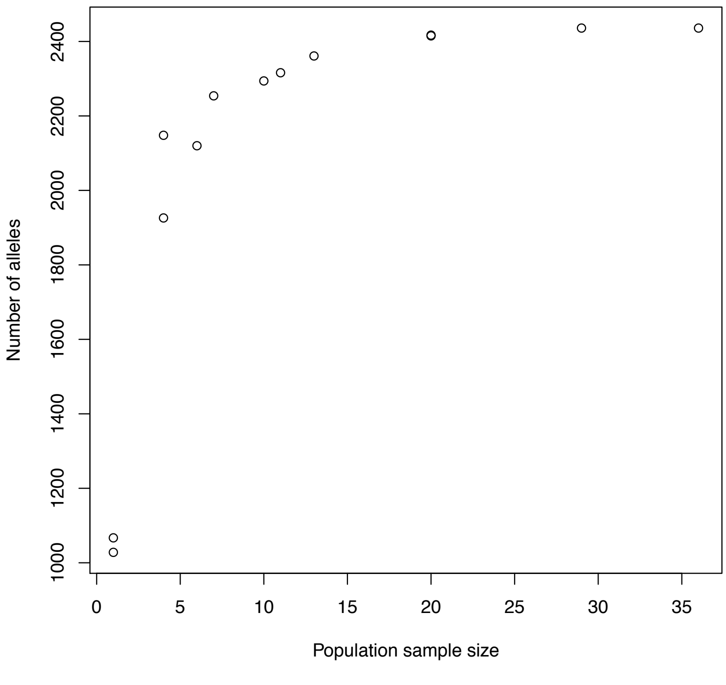

Figure FS3. Maximum likelihood phylogenetic tree. ML tree reconstructed from 1,220 SNPs and 162 different *P. volitans* genotypes. Different branch colours represent localities where genotypes were collected. Specimens are identified by their population code (as in Table 1) followed by an individual number. Only bootstrap support over 40% is represented on the nodes.

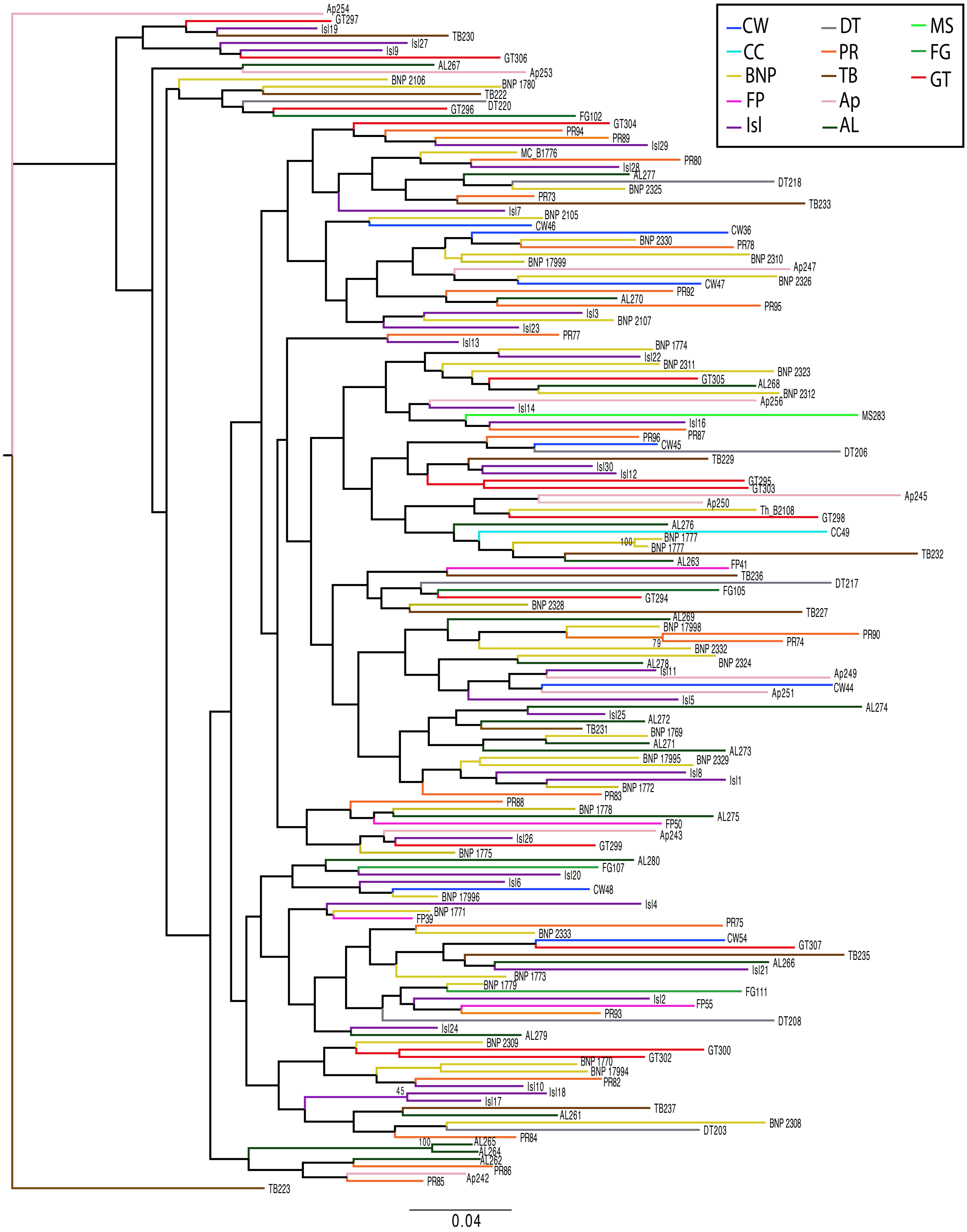

Figure FS4. Structure output for all K values. Values of the mean Log likelihood for each K (top left), optimal number of clusters detected according to the ad hoc statistic ΔK and represented as red circles (Delta K)(top right), and barplots from K=2 to K=8.

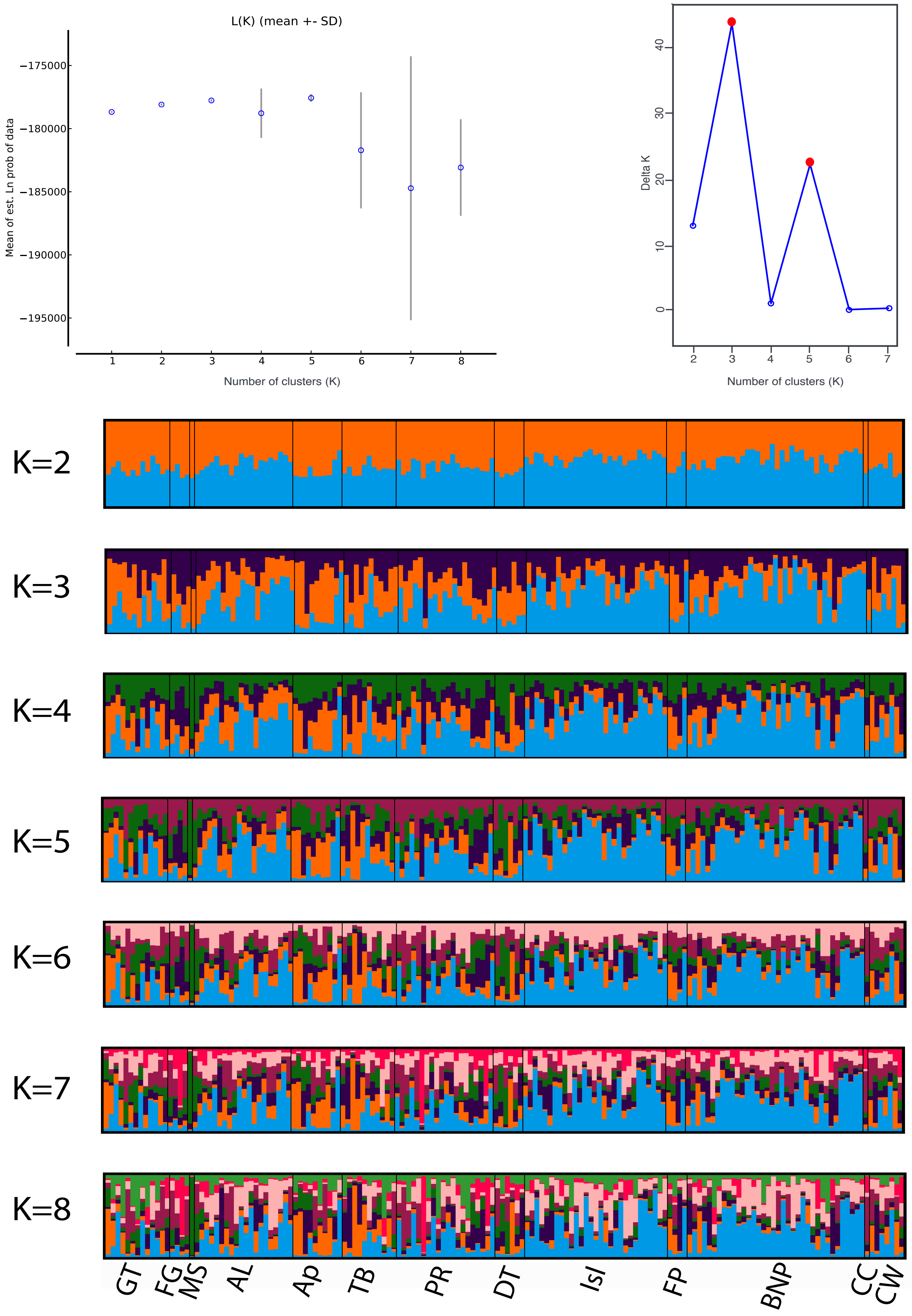

Table TS1. Results of analyses of molecular variance (AMOVAs) of lionfish from 1,207 neutral SNPs. Populations' grouping (Source of variation), degrees of freedom (d.f.), percentage of variation (% Var.), fixation indexes, and *p*-values.

**Source of variation**	**d.f**	**% Var.**	**Fixation index**	***p*-value**
PR and DT within NW Atlantic				
Among groups	1	0	-0.001	0.84
Among populations within groups	11	0	-0.007	1.00
Within populations	149	0	-0.040	0.99
Within individuals	162	100	-0.048	0.99
PR and DT within Gulf of Mexico				
Among groups	1	0	-0.002	0.89
Among populations within groups	11	0	-0.006	1.00
Within populations	149	0	-0.040	0.99
Within populations	162	100	-0.049	0.99
Without PR and DT				
Among groups	1	0	-0.002	0.86
Among populations within groups	11	0	-0.006	1.00
Within populations	125	0	-0.049	0.99
Within populations	136	100	-0.057	0.99
